# Metabolomic and metagenomic analyses elucidate the role of intercropping in mitigating continuous cropping challenges in tobacco

**DOI:** 10.3389/fpls.2024.1447225

**Published:** 2024-12-23

**Authors:** Ming Liu, Rujun Xue, Shuangzhen Jin, Kaiyuan Gu, Jie Zhao, Shuyue Guan, Xiaoyu Xie, Jiaen Su, Longchang Wang

**Affiliations:** ^1^ College of Agronomy and Biotechnology, Southwest University/Engineering Research Center of South Upland Agriculture, Ministry of Education, Chongqing, China; ^2^ Dali Prefecture Branch of Yunnan Tobacco Company, Dali, Yunnan, China; ^3^ Weishan City Branch of Yunnan Tobacco Company, Weishan, Yunnan, China

**Keywords:** metagenomics, tobacco continuous cropping obstacles, rhizosphere soil, ABC transporter pathway, intercropping system, crop mixtures

## Abstract

**Introduction:**

Crop rotation of tobacco with other crops could effectively break the negative impact of continuous tobacco cropping, but the mechanisms of intercropping system effects on tobacco, especially on the rhizosphere, are not clear.

**Methods:**

In this study, we investigated the impact of intercropping system on the diversity and function of tobacco metabolites and microorganisms through metabolomic and metagenomic analyses of the tobacco rhizosphere microenvironment intercropped with maize and soybean.

**Results:**

The results showed that the contents of huperzine b, chlorobenzene, and P-chlorophenylalanine in tobacco rhizosphere soils differed significantly among soybean-tobacco and maize-tobacco intercropping system. Chlorobenzene and P-chlorophenylalanine had the highest relative abundance under the soybean-tobacco intercropping system, and huperzine b had the highest relative abundance in the maize-tobacco cropping system. At the phylum level, the three most dominant strains were the same across all treatments: *Proteobacteria, Actinobacteria*, and *Acidobacteria*, with only minor differences in their abundance, with the fourth most abundant strain in both the tobacco monoculture. KEGG enrichment analysis of the tobacco rhizosphere soil microbiome revealed that intercropping significantly increased the abundance of metabolites in the ABC transporters pathway and up-regulated the *LivK, LivH, Livg, LivM*, and *LivF* genes of the branched-chain amino acid pathway

**Discussion:**

Collectively, our results indicate that the intercropping could enhance the activity of Livs to enhance the ABC transport pathway, and thus improve the transmembrane transport ability of tobacco roots, thus reducing the negative impact of continuous tobacco cropping. At the same time, the maize-tobacco intercropping could promote the production and transportation of phenolic acids, flavonoids, and other bioactive substances in the tobacco root system, which could enhance tobacco adaptation capacity to abiotic stress.

## 1 Introduction

Tobacco (*Nicotiana tabacum* L.) is an important cash crop in China, with an annual planting area of approximately 1.41 million hectares. Yunnan Province, one of the major tobacco-producing areas, has significant problems with continuous tobacco cropping for extended periods of time. Crop rotations and intercropping have been adopted locally to achieve efficient utilization of resources and ecological balance through rational allocation of different crop types (Chen et al., 2022; Wang et al., 2023). Plant rhizosphere secretions and microbial activities together shape the unique microenvironment of the rhizosphere of the crops in intercropping systems. Shi et al. (2023) showed that the composition of rhizosphere secretions, such as organic acids, amino acids, and sugars, varies significantly among crops, which not only directly affect the soil physicochemical properties, but also provide nutrients for the rhizosphere microorganisms, guiding the formation of the microbial community structure (Zhao et al., 2023; Mavrodi et al., 2021; Gautier et al., 2020). Rhizosphere microorganisms produce a range of bioactive substances such as enzymes, antibiotics, and hormones as they carry out their life activities, further regulating the rhizosphere microenvironment (Bhatti et al., 2017; Ahmed and Hijri, 2021).

Metagenomics can reveal the species composition, functional potential, and mutualistic networks of rhizospheric soil microbial communities by analyzing their genome-wide information. Many researchers have utilized metagenomics to investigate species diversity and functional diversity and probe changes in agroecology soil nutrient cycling, biodegradation, and biological nitrogen fixation. Arunrat et al., in a study of maize fields in the Chiang Mai region of Thailand, showed that undisturbed soil over a 5-year period exhibited more stability in bacteria richness and diversity across seasons compared with the soil that was continuously cropped with maize for 5 years (Arunrat et al., 2023). Research investigated the rhizosphere microbiome of three wheat cultivars with high-Zn and three with low-Zn accumulation capacity in a field experiment in calcareous soils and found that most of the rhizosphere-enriched microbes in wheat were common, including many of the previously reported soil Zn-mobilizing microbes (Wang et al., 2021). The bulk of the plant microbiota is concentrated at the subterranean plant root–soil interface. Plant roots secrete metabolites containing primary and secondary low-molecular-weight metabolites, lysates, and slime. These exudates provide nutrients to soil microorganisms and regulate their affinity for the host plant. Metabolomics can accurately measure and reveal the roles of a wide range of small-molecule metabolites in the inter-root environment in physiological processes, signaling, and ecological functions. Based on high-throughput sequencing technology and metabolomics technology, the rhizosphere soil microbial community and metabolic dynamics of healthy banana plants were analyzed during one growth cycle, and it was found that healthy banana rhizosphere soil not only has a stable micro-ecology but also has stable metabolic characteristics. Moreover, rhizosphere soil microorganisms from healthy banana plants have mutually beneficial rather than competitive relationships (Ye et al., 2022).

Metagenomes and metabolomes are closely related through “gene–metabolite–function” interconnections, which, together, shape the unique tobacco rhizosphere microenvironment under different cropping schemes. On the one hand, the gene expression of rhizosphere microorganisms determines their metabolic potential and generates abundant metabolites, which affect the soil nutrient dynamics, pH, redox status, etc. On the other hand, rhizosphere metabolites serve as signaling molecules for microorganism functions and survival, which drive the succession of microbial community structure and function. In this study, we aimed to elucidate the role of intercropping in mitigating the challenges of continuous cropping of flue-cured tobacco by examining the changes in rhizosphere microorganisms and metabolites in intercropping of different crops and to identify the altered metabolic pathways that enhance the response of flue-cured tobacco to abiotic stresses (continuous cropping challenges).

## 2 Materials and methods

### 2.1 Experimental site selection and experimental design

This experiment was conducted in December 2022 in Dali, Yunnan Province, China. In 2023, the average annual precipitation in Dali was 823.3 mm, the average annual temperature was 17.0°C, and the average annual sunshine duration was 2,346.2 h. The experimental site is 2,034 m above sea level. The main physical and chemical properties of the soil were as follows: soil bulk density 1.32 g·cm^−3^, pH 6.51, organic matter content 26.87 g·kg^−1^, total nitrogen content 1.69 g·kg^−1^, total phosphorus content 1.32 g·kg^−1^, and total potassium content 33.61g·kg^−1^.

The field trial was conducted in a randomized block design with three treatments, each with three replicates. Each replicate plot covered an area of 160 m^2^ (20 m * 8 m). All preceding crops in the plots were tobacco. The three treatments were as follows: (1) CK: tobacco monoculture; (2) MT: intercropping of tobacco and maize; (3) ST: intercropping of tobacco and soybean. Regarding the plant materials, the tobacco variety K326, the maize variety Yuntianyu No. 9 (growth period, 95 days), and the soybean variety Yunwan No. 18 (growth period, 100 days) were used in the experiments. The tobacco was planted in May 2023, and the intercropped crop was sown in July. The row spacing of tobacco plants was 120 cm * 50 cm, and the intercropped crops were planted at 10 cm of the tobacco stem base. 15,000 kg/hm^2^ of decomposed fine farm manure, 450 kg/hm^2^ of superphosphate, and 150 kg/hm^2^ of potassium sulfate were applied as basal fertilizer. Topdressing fertilizer was applied before maize/soybean flowering, with 675 kg/hm^2^ of ammonium bicarbonate and 150 kg/hm^2^ urea. Other field management measures were carried out in accordance with local field management measures.

### 2.2 Collection of soil samples

Tobacco rhizosphere soil samples were collected on 18 August 2023, when the middle and upper leaves of the tobacco were harvested. Twenty tobacco plants with similar growth were selected in each treatment, and the soil adhering to the roots was scraped to acquire rhizosphere samples (Li et al., 2019). The rhizosphere soil samples were sieved through a 2.0-mm sieve to remove plant debris and rocks, and 20 plants were combined to form six samples, then immediately sent to Shanghai Majorbio Bio-pharm Technology Co., Ltd with dry ice for metagenomic and metabolomic analysis.

### 2.3 Analysis of metabolites in tobacco rhizosphere

Four biological replicates, 50 mg of the rhizosphere soil sample, were added to a solvent of acetonitrile, methanol, and water (volume ratio 2:2:1). The sample was then vortexed and sonicated three times in an ice-water bath. The supernatant was centrifuged and subjected to UHPLC-Q Exactive (QE) Orbitrap MS analysis. Quality control samples were generated by pooling the supernatant from all samples and detecting soil metabolites via liquid chromatography-tandem mass spectrometry (LC-MS/MS) (Shanghai Majorbio Bio-pharm Technology Co., Ltd), according to the method of Hou et al. (2021).

Chromatographic separation of metabolites was conducted on a Thermo UHPLC-Q Exactive HF-X system equipped with an ACQUITY BEHC 18 column (100 mm * 2.1 mm, 1.7 μm; Waters, USA). The mobile phases consisted of 0.1% formic acid in acetonitrile and 0.1% formic acid in acetonitrile. The sample injection volumes were 2 μL, and the flow rate was 0.4 mL min^−1^. The column temperatures were 40°C. During the analysis period, all of the samples were stored at 4°C. The mass spectrometric data were collected using a Thermo UHPLC-Q Exactive Mass Spectrometer equipped with an electrospray ionization source operating in either positive or negative ion mode.

The raw data were uploaded into Progenesis QI 2.3 (Nonlinear Dynamics, Waters, USA) for peak detection and alignment. The data analyses were performed on an online analysis platform (Majorbio Biotech Co., Ltd., Shanghai, China). The identified metabolites were annotated to the KEGG database (https://www.kegg.jp/). Partial least squares discriminant analysis (PLS-DA) using a supervised clustering method was applied to identify treatment group differences and discern variables for class separation. The variable importance in projection (VIP) values were calculated to identify the contributions of various variables in the PLS-DA model. The metabolites with VIP > 1.0 and *p* < 0.05 (*t*-test) were considered to be differential metabolites (DEMs) (Zhang et al., 2020). Venn diagrams of the DEMs were used to present the number of unique or shared upregulated and downregulated metabolites among the compared treatment groups. A heatmap that combined hierarchical clustering analysis was employed to visualize the expression patterns of the metabolites under different treatments. The pathway enrichment of the DEMs was assessed based on the KEGG database. The top 10 metabolic pathways were presented by bubble charts. The upregulated or downregulated DEMs were classified by the HMDB database (https://hmdb.ca/). The differences in the top 20 DEMs with the highest VIP values between the compared groups were displayed using heatmaps.

### 2.4 Metagenomic analysis of tobacco rhizospheric microorganisms

Total soil DNA from the rhizosphere soil samples was extracted from 500 mg of fresh samples with the Power Soil^®^ DNA Isolation Kit (Qiagen, Hilden, Germany) following the manufacturer’s protocols. After genomic DNA extraction, the quality of the extracted genomic DNA was assessed by 1% agarose gel electrophoresis. Covaris M220 was used to cut genomic DNA into fragments of approximately 350 bp. Then, a PE library was constructed, the self-contiguous segment of the joint was removed by magnetic beads, the library template was enriched by PCR amplification, and single-strand DNA fragments were generated by sodium hydroxide denaturation. One end of each DNA fragment was complementary to the primer sequence and fixed on the chip. The other end was complementary in a random manner with another nearby primer and then amplified by PCR to produce DNA clusters. Then, the DNA amplicon was linearized into a single strand. Illumina sequencing was then performed. Quality control was performed on the raw sequencing data, with the removal of adapters, sequence trimming, and removal of low-quality reads. Then, the optimized sequences were used for sequence assembly and gene prediction, and the obtained genes were annotated and classified in terms of species. Megahit software was used to assemble high-quality reads, and the obtained contigs shorter than 300 bp were discarded. After gene prediction with MetaGene, genes were clustered to remove redundant sequences using CD-Hit at 90% identity and 90% coverage. To perform taxonomic and functional analysis, the genes were compared (BLASTp) against NCBI-nr and KEGG databases using DIAMOND with an e-value cutoff of 10^−5^.

### 2.5 Data analysis

The KEGG online service tool (http://wwwgenomejpy/kaas-bin/kaas_main) was used to annotate the protein with KEGG. Subsequently, the differential proteins were enriched in the corresponding pathway using the KEGG mapper. The heatmap was generated using the pheatmap package in RStudio (R 4.1.2) software, whereas the volcano plot and bubble diagram were generated using theggplot2 package. The data of endogenous hormones were analyzed by SPSS 22.0 (SPSs Institute Inc.) and Origin 8.0 (Origin Lab). The data were analyzed using the online tools of the Majorbio Cloud Platform (https://cloud.majorbio.com/page/tools/). Sequence data were deposited in the NCBI SRA database under the accession number SUB14433325.

## 3 Results and analysis

### 3.1 Metabolome analysis of tobacco root microenvironment under different intercropped crops

#### 3.1.1 Metabolite analysis

**Figure 1:**
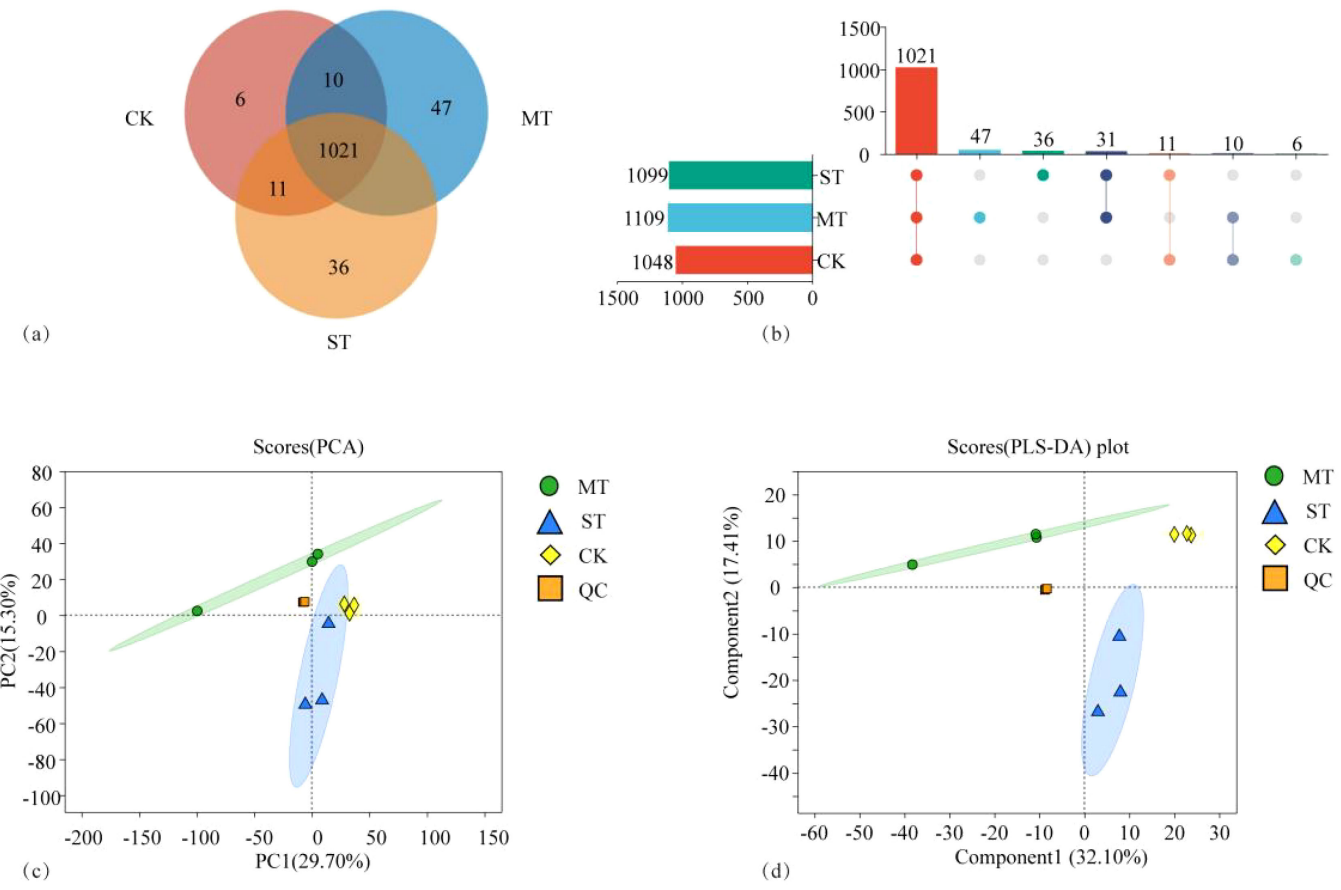
Venn diagram **(A)**, UpSet diagram **(B)**, PCA **(C)**, and PLS-DA diagram **(D)** of tobacco rhizosphere metabolites affected by different intercropped crops.

**Figure 1** shows the comparative analysis of metabolites in tobacco roots under different treatments. As shown in **Figures 1A, B**, a total of 1,162 metabolites were identified by the metabolome analysis, among which 1,021 metabolites were shared under the three treatments. There were 1,048 metabolites in CK, 1,109 in MT, and 1,099 in ST, and the metabolites identified in CK were fewer than those in the other two treatments, specifically 5.82% and 4.87% fewer than MT and ST, respectively. There were 1,031 shared metabolites in CK vs. MT, 17 specific to CK and 78 specific to MT. In CK vs. ST, there were 1,032 shared metabolites, 16 specific to CK and 67 specific to ST. There were 1,052 shared metabolites in MT vs. ST, with 57 MT-specific metabolites and 47 ST-specific metabolites. In the CK vs. MT vs. ST comparison, there were 6 metabolites uniquely identified in CK, 47 unique metabolites in MT, and 36 unique metabolites in ST. Principal component analysis (PCA) and PLS-DA analyses were performed on all metabolites identified under the treatments (**Figures 1C, D**), revealing that the overall metabolite composition under the three treatments, CK, MT, and ST, was significantly different, and the variation among samples within each treatment group was low. The results of this study suggest that intercropping with other crops could improve the richness of metabolites in the rhizosphere of tobacco.

**Figure 2:**
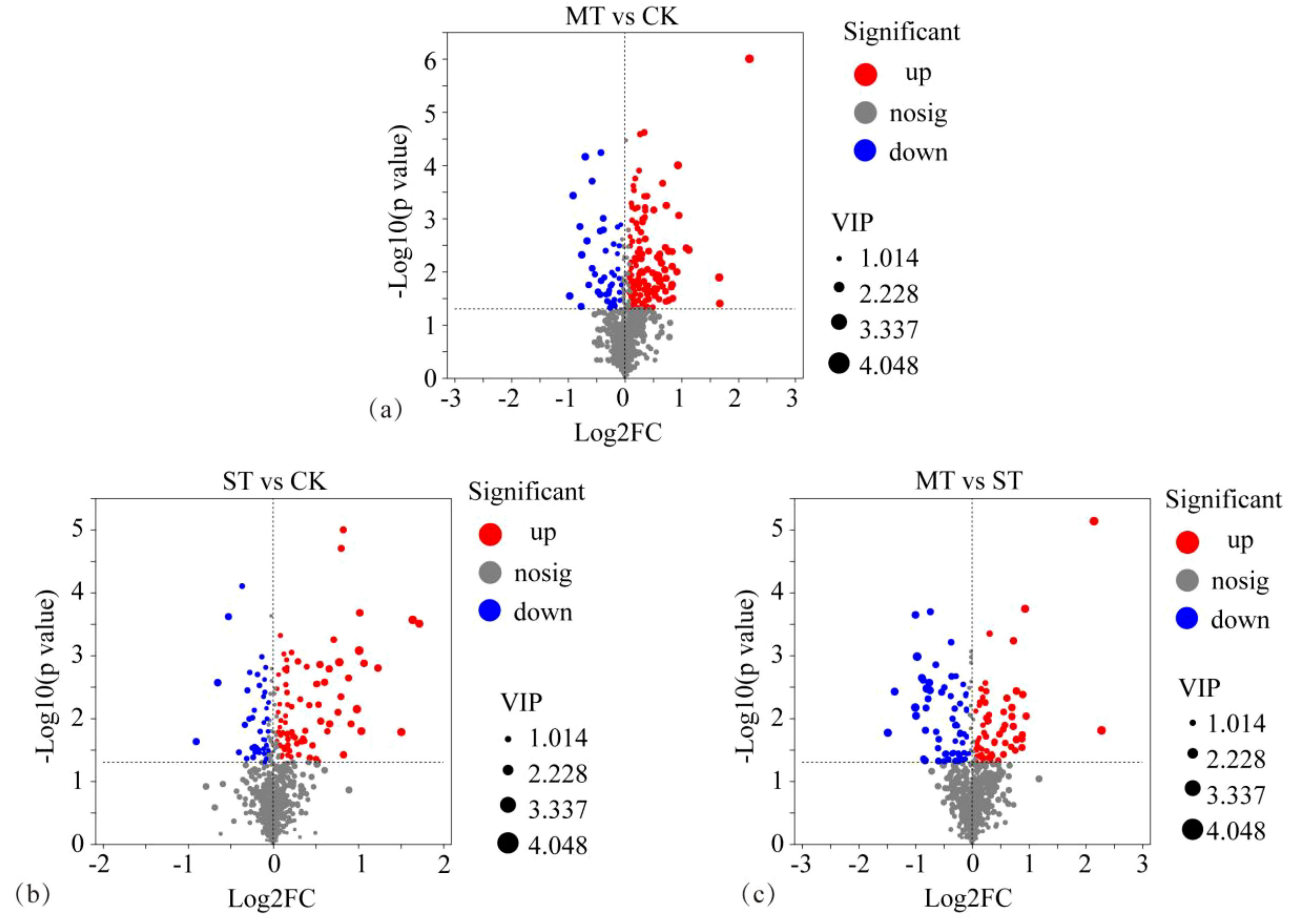
Volcanic map of different rhizosphere metabolites of tobacco from different intercropping crops. **(A)** CK vs. MT; **(B)** CK vs. ST; **(C)** ST vs. MT. The horizontal coordinate is the multiple change value of the difference in metabolite expression between the two groups, that is, log2FC; the vertical coordinate is the statistical test value of the difference in metabolite expression, that is, the value of −log10(*p*-value). Both the horizontal and vertical values were logized. Each dot in the diagram represents a specific metabolite, and the size of the dot represents the VIP value. By default, red dots represent significantly upregulated metabolites, blue dots represent significantly downregulated metabolites, and gray dots represent non-significantly differentiated metabolites.

**Figure 3:**
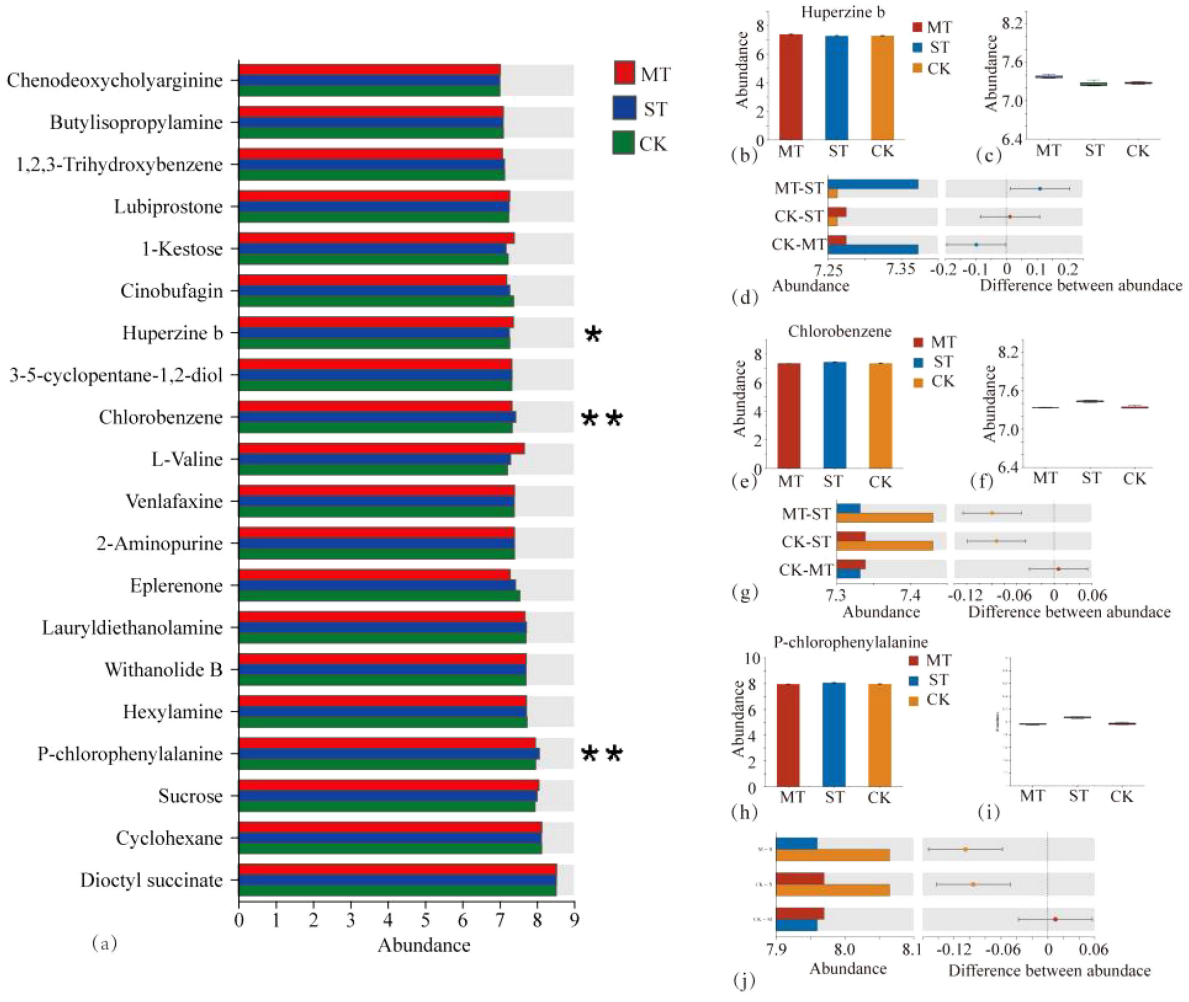
One-way ANOVA analysis of tobacco rhizosphere metabolites by different intercropping crops and relative abundance analysis of three metabolites with significant differences in different treatments. **(A)** The *Y*-axis represents the name of metabolites, the *X*-axis represents the average relative abundance of metabolites in different groups, and columns of different colors represent different groups; The far right is the *p*-value, *0.01 < p ≤ 0.05, **0.001 < p ≤ 0.01. Content of different metabolites in different treatments **(B, C, E, F, H, I)**. In **(D, G, J)**, the *X*-axis of the left column graph represents the average relative abundance of metabolites in different groups, and the ordinate represents the group category of pin-wise comparison. Different colors represent different groups. The middle region is within the set confidence interval, and the values corresponding to the dots represent the difference of the average relative abundance of metabolites in the two groups. The color of the dots is displayed as the color of the group in which the abundance of metabolites accounts for a larger proportion, and the type I interval on the dots is the upper and lower limit of the difference.

By comparing CK, MT, and ST, the metabolites with significant differences between treatments were identified and compared (**Figure 2**). These metabolites with significant differences and stable high accumulation are likely to participate in important metabolic pathways and perform extremely important biological functions. The results revealed 93 significantly upregulated metabolites, 26 significantly downregulated metabolites, and 576 metabolites with non-significant differences in CK vs. MT (**Figure 2A**). In CK vs. ST, there were 86 significantly upregulated metabolites, 41 significantly downregulated metabolites, and 958 metabolites with non-significant differences (**Figure 2B**). In ST vs. MT, there were 70 significantly upregulated metabolites, 66 significantly downregulated metabolites, and 949 metabolites with non-significant differences (**Figure 2C**). The distribution of tobacco rhizosphere metabolites under different intercropped crops was compared with one-way ANOVA analysis (**Figure 3**) to identify significant differences, and then *post-hoc* tests were conducted on the metabolites with differences. Treatment groups with differences were identified (metabolites with significant differences between treatments were screened according to *p* < 0.05). In tobacco rhizosphere metabolites, we found that the average relative abundance of huperzine b, chlorobenzene, and P-chlorophenylalanine was significantly different under the three treatments. The *p*-value for chlorobenzene and P-chlorophenylalanine was <0.01. Further analysis of these three metabolites showed that chlorobenzene and P-chlorophenylalanine had the highest relative abundance in ST, while huperzine b had the highest relative abundance in MT. The results of this study showed that most of the differentially expressed metabolites in the rhizosphere of tobacco were significantly upregulated compared with tobacco monocultural cropping. These upregulated metabolites might represent the changes in rhizosphere secretory activities of tobacco, which were potential factors for breaking the continuous cropping challenges of tobacco.

#### 3.1.2 KEGG pathway enrichment analysis

**Figure 4:**
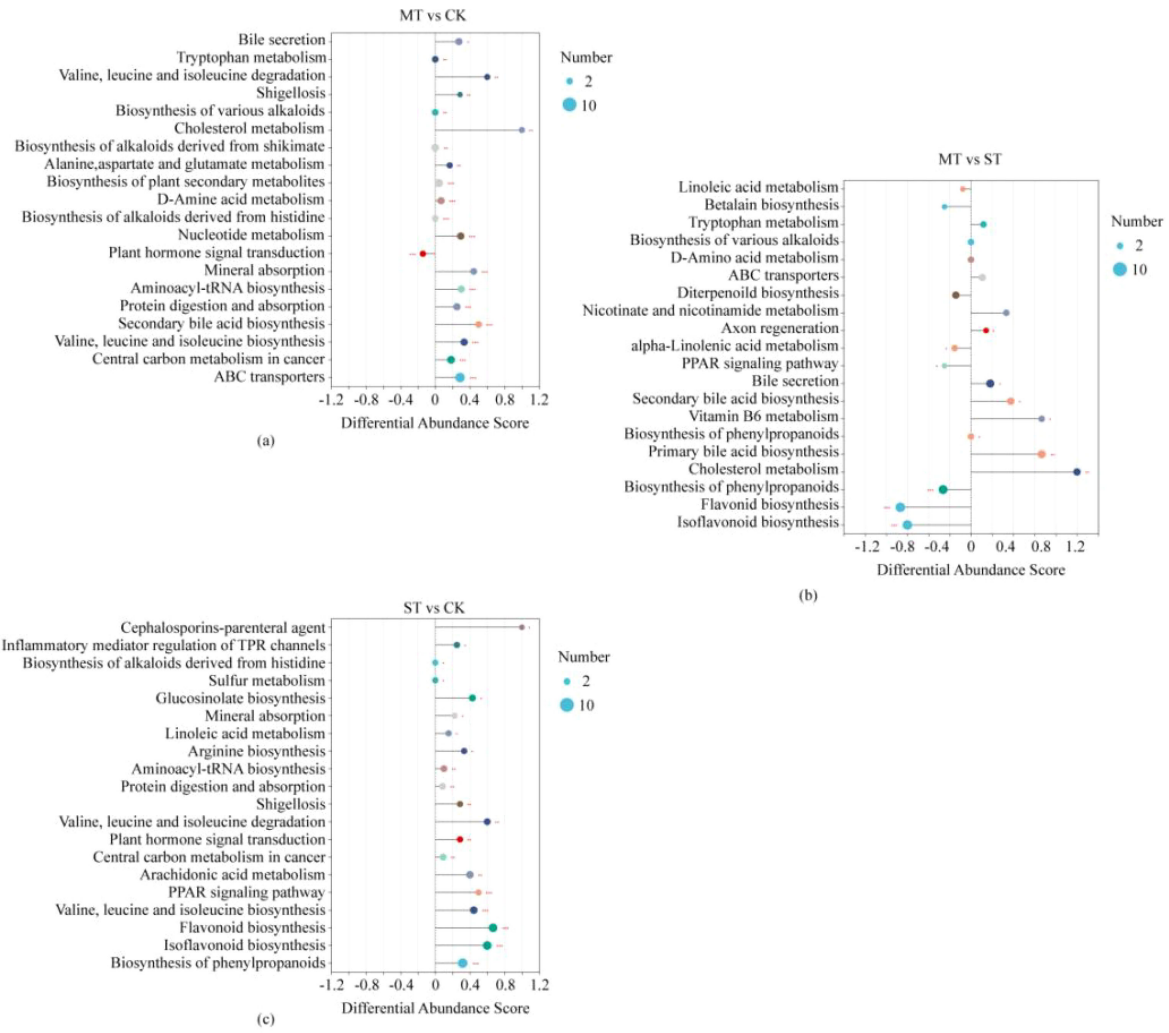
Enrichment analysis of KEGG pathway for tobacco rhizosphere metabolites by different intercropping crops. **(A–C)** are the KEGG pathway difference score graphs, in which the horizontal coordinates represent the difference abundance scores (DA Score), and the vertical coordinates represent the KEGG metabolic pathway names. The DA Score reflects the metabolic pathway of all the metabolites as a whole; a score of 1 indicates that the expression trend of all annotated differential metabolites in the pathway is upregulated, −1 indicates that the expression trend of all annotated differential metabolites in the pathway is downregulated, and the length of the line segment indicates the absolute value of the DA Score. The size of the dots indicates the number of annotated differential metabolites in the pathway.

The enrichment of specific metabolites in the rhizosphere of tobacco under different intercropped crops was assessed. A hypergeometric distribution algorithm was used to obtain the significantly enriched pathways among the metabolites based on the metabolite concentration. The BH method was used to correct the *p*-value, and when the corrected *p*-value was <0.05, it was considered that this pathway was significantly enriched (**Figure 4**). As shown in **Figure 4A**, a total of 65 target metabolites in MT vs. CK were annotated into the KEGG pathway. The plant hormone signal transduction:EIP pathway was enriched with the highest ratio of the metabolite number annotated to the pathway versus the background number, while the ATP-binding cassette (ABC) transporters:EIP pathway was enriched with the highest degree of significance. At the same time, most metabolites were enriched in the ABC transporter pathway. The plant hormone signal transduction pathway was the only pathway whose metabolites tended to be downregulated, while metabolites in the sterol metabolism pathway were mostly upregulated. As shown in **Figure 4B**, a total of 48 target metabolites were annotated to KEGG pathways in ST vs. CK. Among them, the PPAR signaling pathway:OS pathway was significantly enriched and had the highest ratio of pathway-specific metabolite number compared to the background metabolite number. In contrast, the biosynthesis of phenylpropanoids:M pathway was the most significantly enriched pathway and had the highest number of metabolites enriched to this pathway. All pathways in ST tended to be upregulated relative to the overall metabolite profile of CK. As shown in **Figure 4C**, a total of 45 target metabolites were annotated to KEGG pathways in MT vs. ST. Among them are the enriched cholesterol metabolism OS pathway and the PPAR signaling pathway: OS pathway had the highest ratio of pathway-specific metabolite number compared to the background metabolite number. The Isoflavonoid biosynthesis:M pathway was the most significantly enriched and, together with the Flavonoid biosynthesis pathway, had the highest number of metabolites enriched. Unlike MT vs. CK and ST vs. CK, the overall expression of metabolic pathways in MT vs. ST was inconsistent, with 9 of the 20 enriched metabolic pathways being mostly upregulated, 8 being mostly downregulated, and 3 remaining unchanged. The results of this study showed that the enriched pathways of the specific metabolites in the rhizosphere of tobacco under MT and ST treatments were significantly different from those in CK, and most of them were upregulated, and only one pathway was downregulated compared with CK.

### 3.2 Metagenomic analysis of tobacco root microenvironment under different intercropped crops

#### 3.2.1 Analysis of microbial diversity of tobacco rhizosphere under different intercropped crops

Alpha diversity analysis was performed to assess the richness and diversity of tobacco rhizosphere microbial communities under different intercropped crops. As shown in **Table 1**, the OTU number under the three treatments differed but not significantly (*p* > 0.05), but there were significant differences in Chao1, Shannon, and Shannon evenness indices. The Chao1 index in MT was significantly higher than that in CK and ST, being 4.06% and 2.80% higher than that in CK and ST, respectively. The Shannon index was significantly higher in ST than in MT, by 8.56%. The Shannon evenness index exhibited the same trend as the Shannon index, being significantly higher in ST than MT by 8.33%. The Chao1 index reflects the community richness of tobacco rhizosphere microorganisms, the Shannon index reflects the community diversity, and the Shannon evenness index reflects the community evenness. Based on the results of this study, the community richness of tobacco rhizosphere microorganisms was the highest in MT. In contrast, the diversity and community evenness of the tobacco rhizosphere microorganisms were significantly higher in ST than in MT and CK.

**Table 1:** Alpha diversity analysis of tobacco rhizosphere microorganisms.

Sample	Unique number	Chao	Shannon	Shannoneven
CK	997,675.66 ± 45,445.29 a	16,638.33 ± 250.97 b	5.63 ± 0.07 ab	0.57 ± 0.01 ab
MT	957,042.66 ± 45,445.29 a	17,343.00 ± 231.72 a	5.37 ± 0.28 b	0.55 ± 0.02 b
ST	941,450.66 ± 45,445.29 a	16,857.00 ± 311.09 b	5.83 ± 0.15 a	0.60 ± 0.01 a

Different letters indicate significant differences between treatments (*p* < 0.05).

#### 3.2.2 Composition analysis of tobacco rhizosphere microorganisms and their functional pathways under different intercropped crops

**Figure 5:**
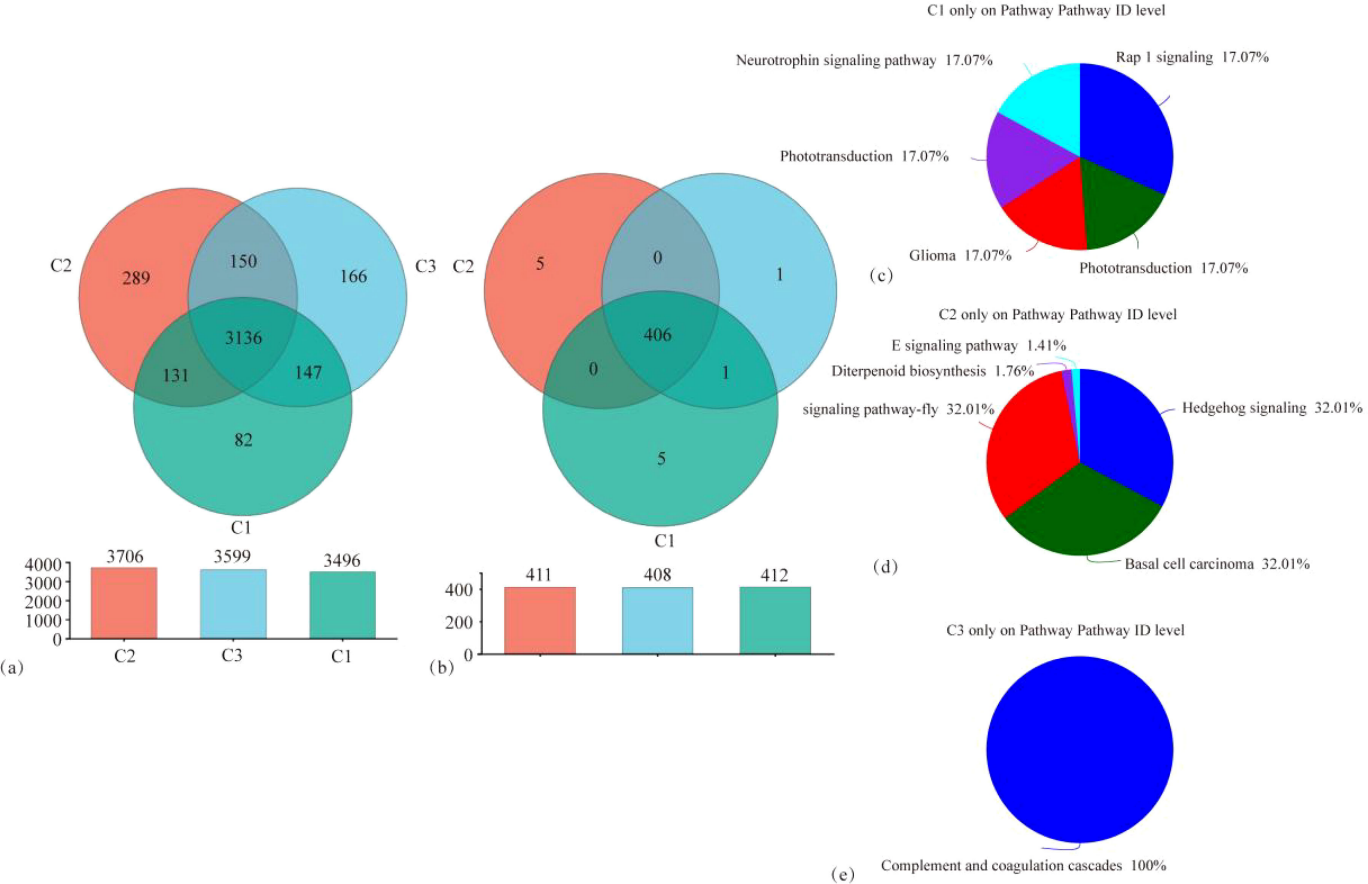
Venn diagram of tobacco rhizosphere microbial species abundance distribution **(A)**, KEGG pathway functional distribution **(B)**, and pie charts of KEGG pathway functional distribution **(C–E)** specific to each treatment.

Species annotation and taxonomic comparisons of tobacco rhizosphere microorganisms under different treatments were carried out using the NR database, and functional pathways were identified and compared using the KEGG database. The distribution of tobacco rhizosphere microorganisms and functional pathways under different treatments is shown in **Figure 5**. As shown in **Figure 5A**, 4,101 OTUs were identified in the three treatments, of which 3,136 OTUs were shared across all treatments. The number of microbial OTUs unique to MT and ST was significantly higher than that of CK. Specifically, the number of OTUs unique to MT was 252.43% higher compared to CK, and the number of OTUs unique to ST was 102.43% higher compared to CK. As shown in **Figure 5B**, 418 functional pathways were identified across the three treatments, of which 3,406 OTUs were shared. Five functional pathways were unique to MT and CK. In comparison, only one functional pathway was unique to ST. In analyzing these 11 specific functional pathways, we found that the five pathways unique to CK were the Neurotrophin signaling pathway, the Phototransduction–fly pathway, the Glioma pathway, the Phototransduction pathway, and the Rap1 signaling pathway. The five pathways unique to MT were the AGE-RAGE signaling pathway in diabetic complications pathway, the Diterpenoid biosynthesis pathway, the Hedgehog signaling pathway, the Hedgehog signaling pathway–fly pathway, and the Basal cell carcinoma pathway, whereas the pathways specific to S are Complement and coagulation cascades pathway. The results of this study showed that the CK treatment had the fewest identified microorganisms and, at the same time, had the fewest unique microbial species compared to MT and ST treatments. However, for KEGG pathways, the least number of unique pathways were identified in ST.

**Figure 6:**
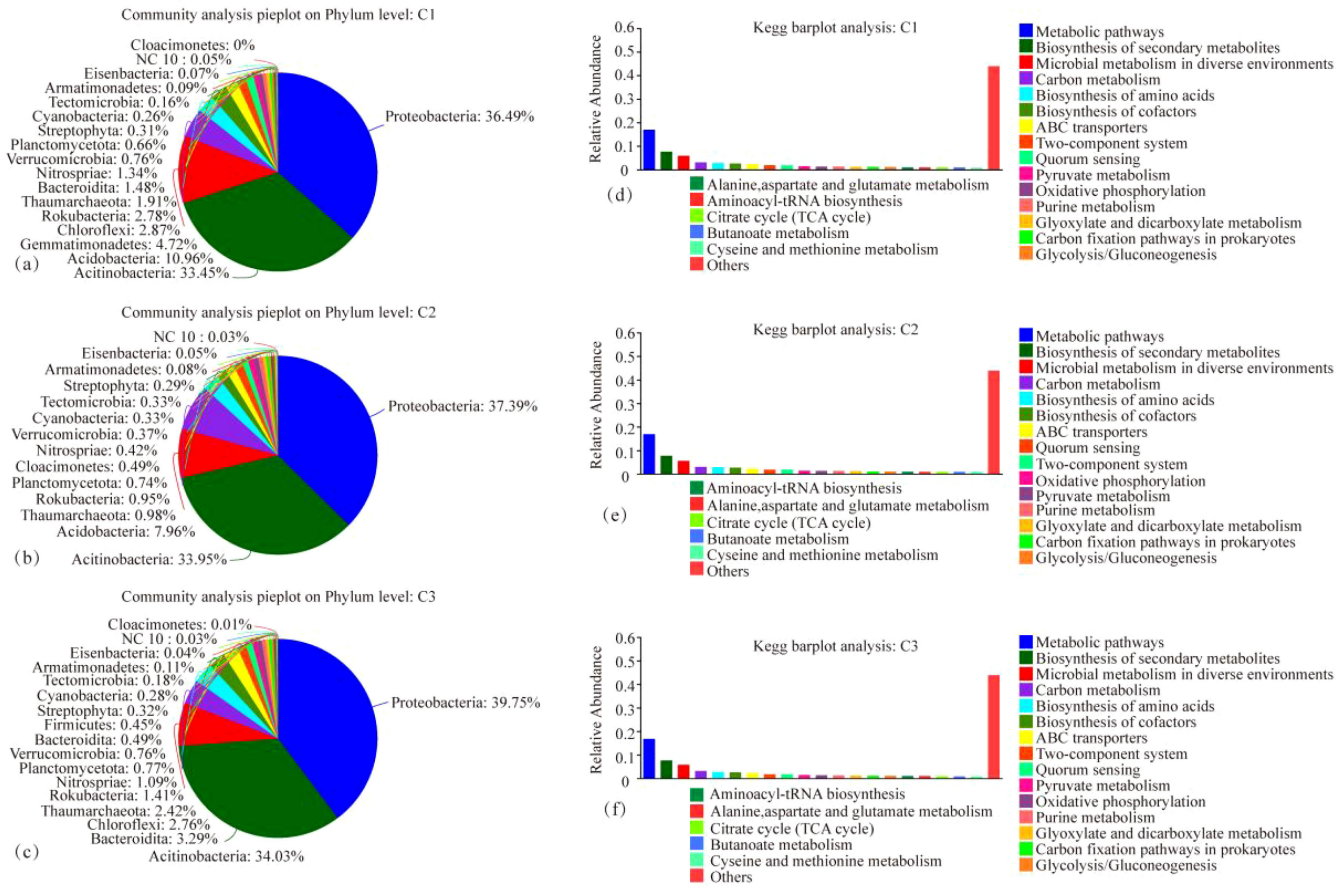
Microbial species composition maps at the phylum level **(A–C)** and KEGG functional pathways bar graphs **(D–F)** under different intercropped crops.

**Figure 7:**
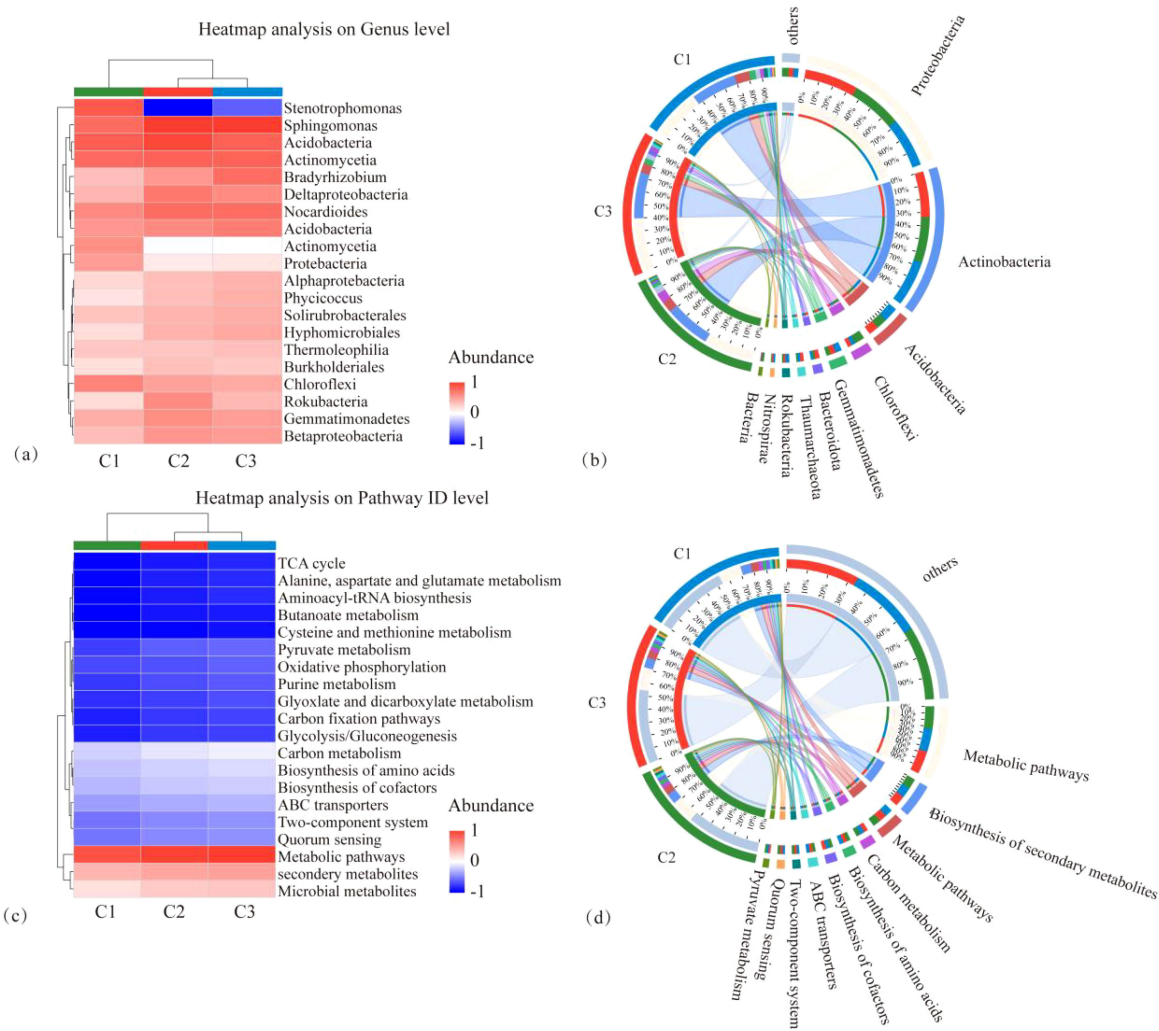
Heatmap of microbial species composition at the phylum level **(A)**, Circos plot **(B)**, KEGG functional pathway heatmap **(C)**, and Circos plot **(D)** under different intercropped crops.

The microbial species composition and annotated KEGG pathways of each treatment, CK, MT, and ST, are shown in **Figure 6**. At the phylum level, the top three dominant phyla were the same in each treatment: *Proteobacteria, Actinobacteria*, and *Acidobacteria*. Only the abundance of individual phyla was slightly different. The fourth phylum in abundance exhibited differences between the treatments, specifically *Gemmatimonadetes* in both CK and ST and *Chloroflexi* in MT. Regarding the KEGG pathway analysis, the top five KEGG pathways in all three treatments were Metabolic pathways, Biosynthesis of secondary metabolites, Microbial metabolism in diverse environments, Carbon metabolism, and Biosynthesis of amino acids. As illustrated in the heatmap analysis of microbial species and KEGG pathway analysis (**Figures 7A, C**), CK and ST were more similar. Meanwhile, the Circos plot also showed that the proportion of *Proteobacteria*, based on the NR database annotation, was 36%, 40%, and 37% in CK, ST, and MT, respectively; the proportion of *Actinobacteria* was 33%, 34%, and 34% in CK, ST, and MT, respectively; and the proportion of *Acidobacteria* was 11%, 7%, and 8% in CK, ST, and MT, respectively (**Figures 7B, D**). In contrast, there was no significant difference in the annotated KEGG pathways among treatments. The results of this study showed that the dominant top three species were the same in all treatments, which were *Proteobacteria*, *Actinobacteria* and *Acidobacteria*. There are only subtle differences in the abundance of the individual species. At the same time, the top five KEGG functions under the three treatments were Metabolic pathways, Biosynthesis of secondary metabolites, Microbial metabolism in diverse environments, Carbon metabolism, and Biosynthesis of amino acids. These results indicated that there were no significant changes in the dominant species and functions of different intercropping crops, but there were slight differences in the proportion of microbial abundance and functions.

#### 3.2.3 Difference analysis of functional pathways of tobacco rhizosphere microorganisms by different intercropping crops

**Figure 8:**
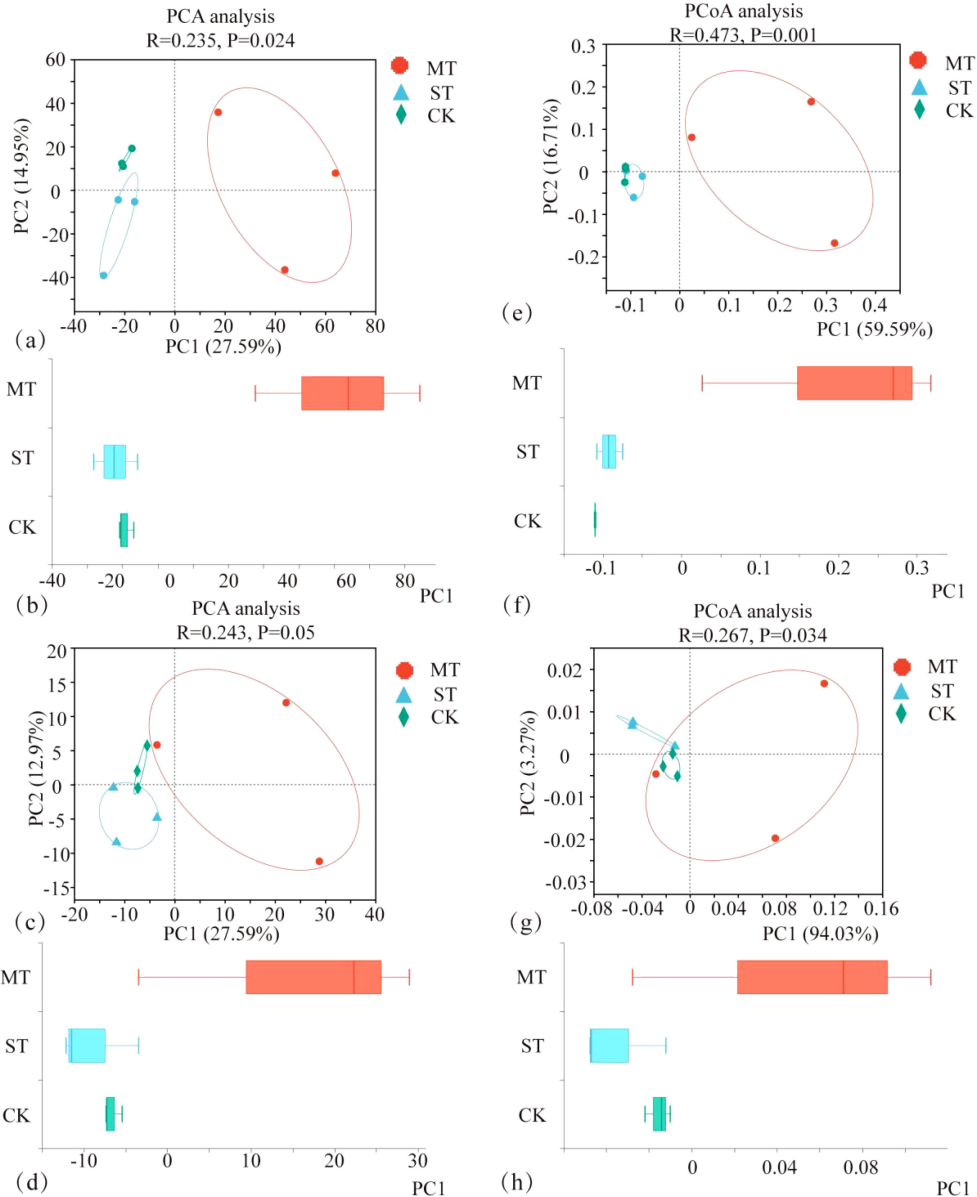
PCA of tobacco rhizosphere microbial species **(A, B)**, PCA of KEGG functional pathways **(C, D)**, PCoA of tobacco rhizosphere microbial species **(E, F)**, and PCoA of KEGG functional pathways **(G, H)** under different intercropped crops.

**Figure 9:**
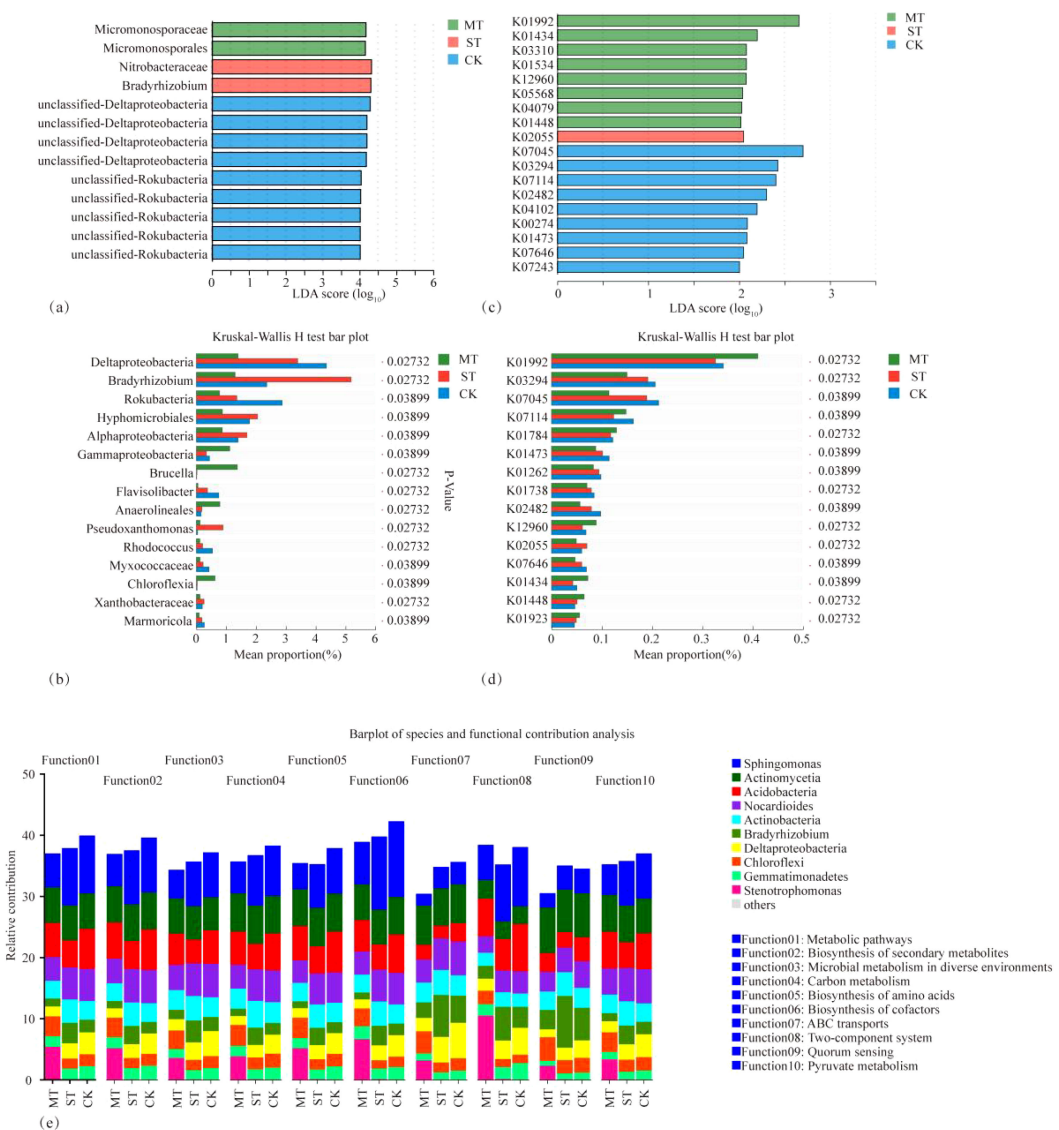
Analysis of differences in LEfSe of microbial species in tobacco rhizosphere under different intercropped crops **(A)**, test of variance bar chart **(B)**, analysis of differences in LEfSe of microbial KEGG functional pathways **(C)**, test of variance bar chart **(D)**, and analysis of the contribution of each species to the different functional pathways identified in the analysis **(E)**.

The PCA and principal coordinate analysis (PCoA) of tobacco rhizosphere microbial species and KEGG functional pathways under different intercropped crops revealed that the microbial community of MT was clearly separated. In contrast, ST and CK were clustered closer. Thus, the degree of similarity between ST and CK was greater (**Figure 8**). In the analysis of LEfSe differences in microbial species of tobacco rhizosphere under different intercropped crops, it was found (**Figure 9A**) that more microorganisms had a greater influence on the differences observed. When the LDA threshold was >4, two species were identified, *Micromonosporaceae* and *Micromonosporales* in MT and *Nitrobacteraceae* and *Bradyrhizobium* in ST. In CK, more microbial species, specifically nine, had a greater influence on the differences observed, of which the two with the highest influence were *Deltaproteobacteria* and *Candidatus_Rokubacteria*. Significant differences in abundance were observed when comparing the abundance of the same microorganisms under different treatments (**Figure 9B**). Among the six phyla with the most significant differences, *Deltaproteobacteria* and *Candidatus_Rokubacteria* were both significantly more abundant in CK than in MT and ST; *Bradyrhizobium* and *Hyphomicrobiales* had significantly higher abundance in ST than in CK and MT; *Gammaproteobacteria* and *Brucella* had significantly higher abundance in MT than in CK and ST. Analyzing the differences in KEGG functional pathways of the tobacco rhizosphere microorganisms under the different crop combinations, it was found (**Figure 9C**) that most of the functions that had a greater impact on the differences observed were identified in MT and CK. When the LDA threshold was >2, 18 pathways that had a greater effect on the differences between treatments were identified, of which 8 pathways were in MT and 9 were in CK. In contrast, only one pathway in ST had a significant impact on the differences between treatments. When analyzing the difference in mean relative abundance of the same pathways under different treatments, it was found (**Figure 9D**) that the vast majority of pathways contributing to the differences were concentrated in the CK treatments, such as K03294, K07045, K07114, K01473, and K01262. Pathways that were identified in the MT treatments were K01992, K01784, K12960, K01434, and K01448. The rhizosphere microbial community contribution in the dominant KEGG pathways was analyzed (**Figure 9E**). Based on their total abundance, microorganisms involved in the top 10 dominant functions mainly included *Sphingomona*s, *Actinomycetia*, *Acidobacteria*, and *Nocardioides*. These four phyla accounted for more than 20% of each pathway and more than 25% of the metabolic pathways. The microorganisms under different treatments contributed differently to the differential KEGG pathways; for example, in the MT treatment, *Stenotrophomonas* had a high contribution in each function. It even reached 10.46% in the intercropping treatments, while *Stenotrophomonas* had almost no contribution to ST and CK. Meanwhile, *Bradyrhizobium* had a large contribution in all KEGG functional pathways in ST but less in MT and CK. The results of this study showed that *Stenotrophomonas* had a high contribution under MT treatment, while its contribution was almost zero in the other treatments. *Bradyrhizobium*, a kind of nitrogen fixing bacteria, contributed significantly more under ST treatment than other treatments.

### 3.3 Joint-omics analysis of the impact of different intercropped crops on the tobacco rhizosphere microenvironment

**Figure 10:**
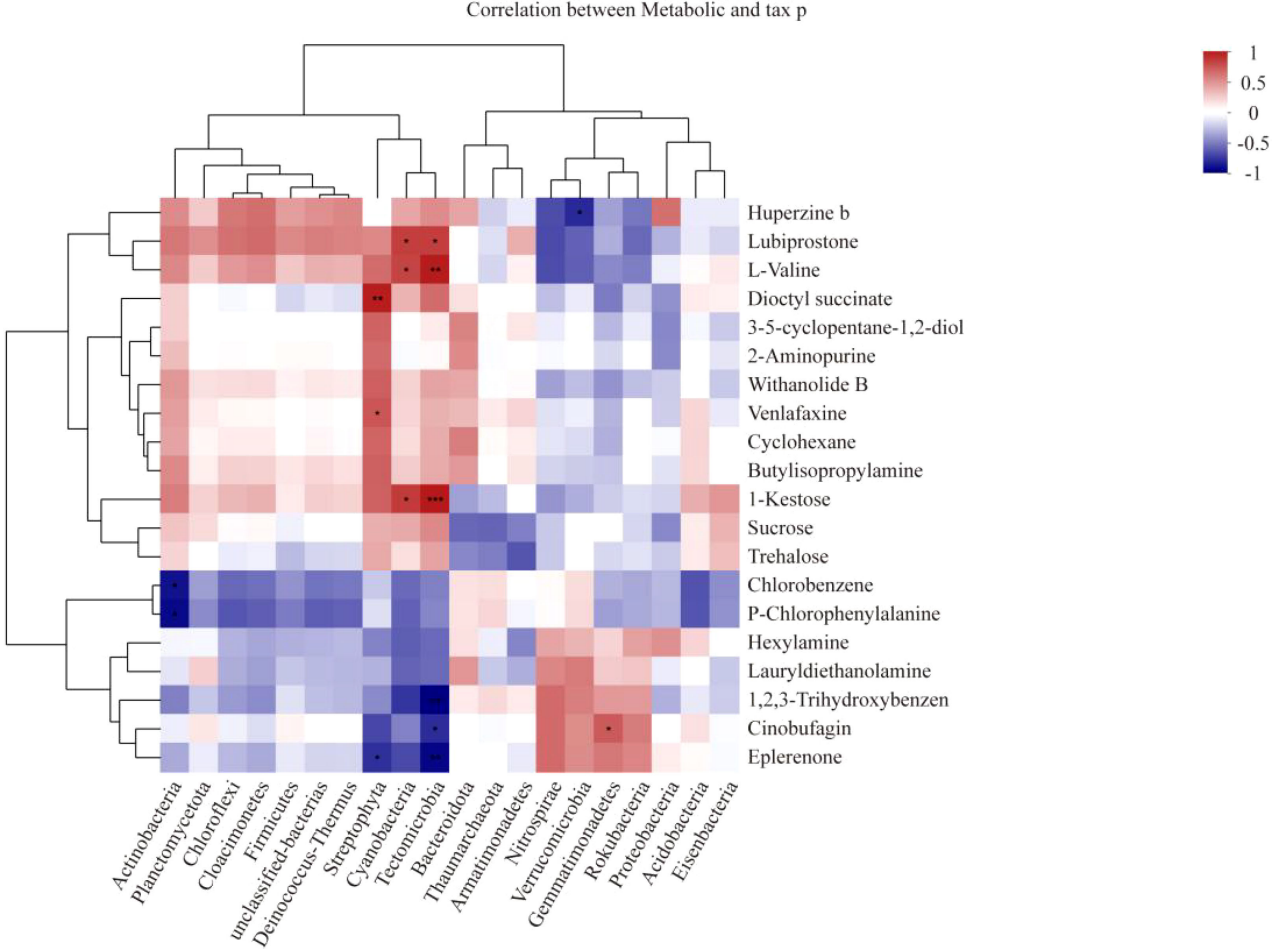
Heatmap of association analysis of tobacco rhizosphere microbial metagenomes and metabolomes under different intercropped crops. *0.01 < p ≤ 0.05, **0.001 < p ≤ 0.01, ***p ≤ 0.001.

**Figure 11:**
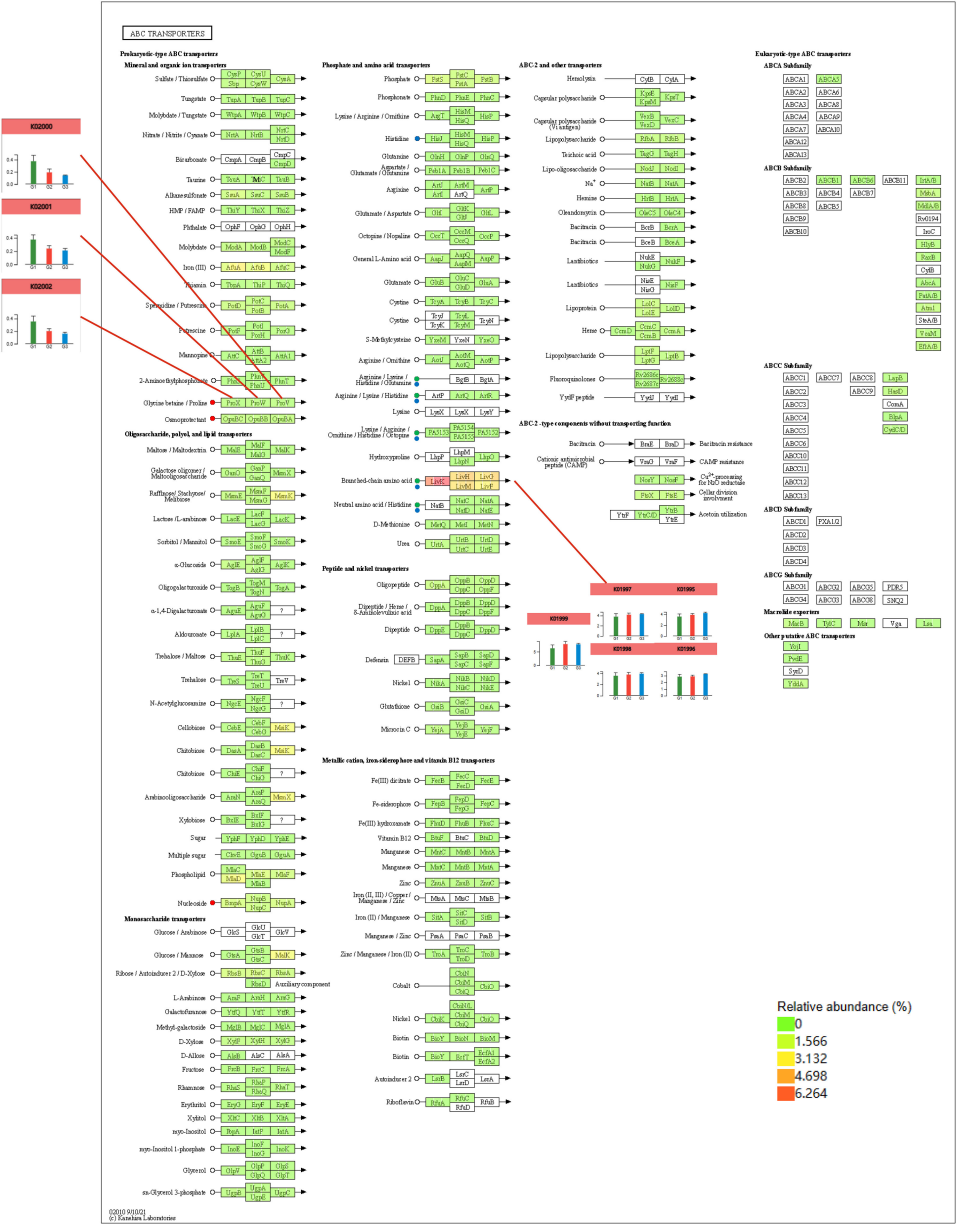
Combined metabolomics and metagenomics analysis of the ABC transporter pathway under different treatments.

In the joint analysis of the metagenome and metabolome of tobacco rhizosphere microorganisms under different intercropped crops (**Figure 10**), it was found that the *Streptophyta, Cyanobacteria*, and *Candidatus Tectomicrobia* metabolites, Kestose, and Valine, were strongly correlated. ABC transporters were enriched to most of the metabolites in MT vs. CK and ST vs. CK. Therefore, we further analyzed the ABC transporters (**Figure 11**) and found that in the ABC transporters pathway, there were two metabolites upregulated in MT compared to CK, which were Betaine and Adenosine; four metabolites were downregulated, which were L-histidine, L-arginine, L-valine, and L-alanine. There were no upregulated metabolites in ST compared to CK, while two metabolites were downregulated: L-histidine and L-valine. As shown in **Figure 11**, the downregulated genes in both MT and ST included mainly phosphate and amino acid transporters. In this pathway, the abundance of *LivK, LivH, Livg, LivM*, and *LivF* genes of the branched-chain amino acid pathway was significantly higher than that of other genes. Comparative gene expression analysis of these five genes under different treatments revealed that the expression of all five genes under MT (green) was slightly lower than the other two treatments. Meanwhile, *LivH, Livg, LivM*, and LivF exhibited higher expression in CK treatment. The genes upregulated in MT compared to CK mainly included mineral and organic ion transporters. Among them, the gene expression of *ProX, ProW*, and *ProV* involved in Glycine/proline metabolism showed the same trend as above with MT > ST > CK.

## 4 Discussion

### 4.1 Differences in rhizosphere metabolites between tobacco and different intercropped crops

The intercropping of tobacco with other crops has been a proven, cost-effective cultivation method (Zhou et al., 2023). In intercropping, root secretions affect the root microenvironment of both crops due to the contact between the root systems of the two crops (Zhou et al., 2023). Tobacco intercropping with different crops may affect the chemical and metabolite composition in the rhizosphere soil. Root secretions of different plants contain various organic substances (Javed et al., 2018) such as phytohormones, secondary metabolites, and carbohydrates (Chen and Liu, 2024; Backer et al., 2018; Hama et al., 2022). Cultivating tobacco with other crops may alter the types and levels of these rhizosphere chemical compounds and metabolites, thereby affecting microbial activity, nutrient cycling, and plant growth in the soil. This study showed significant differences in tobacco rhizosphere metabolites under different intercropped crop combinations. Both tobacco–maize and tobacco–soybean intercropping increased the amount of tobacco rhizosphere metabolites to varying degrees compared to no intercropping. A two-by-two comparison of the three treatments revealed that both MT and ST had more significantly upregulated metabolites than CK. This is in line with previous studies. In a study by Sun et al. (2022), long-term field measurements showed that maize–faba bean intercropping increased crop yield, bacterial richness, and the Shannon index compared to monocropping. Microbial activity alters the metabolites in the soil rhizosphere microenvironment. Tang et al. (2022) directly showed that adenine and adenosine were detected in the rhizosphere soil, and their levels were increased in the intercropping treatment, which was mainly related to enhanced purine metabolism in root and rhizosphere soils in the sugarcane–peanut intercropping system. This study showed that chlorobenzene and P-chlorophenylalanine had the highest relative abundance in ST, while huperzine B had the highest relative abundance in MT. Zhang and Tang (2000) showed that huperzine B, a novel acetylcholinesterase inhibitor, attenuates hydrogen peroxide-induced injury in cells.

In this study, we analyzed the enriched KEGG pathways among tobacco rhizosphere metabolites under different intercropping combinations. Overall, almost all the pathways tended to be upregulated in both MT and ST, compared with CK, suggesting that intercropping with other crops enhances the metabolic activities in the tobacco rhizosphere microenvironment. Only the plant hormone signal transduction pathway tended to be downregulated. In this metabolic pathway, three metabolites were enriched, with N-jasmonoylisoleucine and abscisic acid exhibiting reduced abundance. N-jasmonoylisoleucine, as an important “stress hormone” in plants, plays an essential role in resistance to biotic and abiotic stresses. Hazman et al. (2019) showed, through phylogenetic and gene expression analysis, that a mimic of insect chewing or seedling exposure to salt triggers jasmonate metabolism and signaling. Both treatments induced specific transcriptional changes, along with the accumulation of JA-Ile and a complex array of oxidized jasmonate catabolites. ABC transporters, a superfamily of membrane proteins that are widely distributed in both prokaryotes and eukaryotes with a wide range of functions, has the ability to utilize the energy generated by ATP hydrolysis for transmembrane transport of substrates against the concentration gradient. Yang et al. (2023) showed that the maize ABC transporter *ZmMRPA6* confers cold and salt stress tolerance in plants. The more intricate an organism’s function, the greater the need for ABC transporter-related functions. Compared with tobacco monoculture, the introduction of another crop into an intercropping system led to complex changes in the inter-root microenvironment of tobacco, and many key physiological processes, such as the secretion of epidermal lipids, the transport of phytohormones, the abundance of vesicles for toxic substances, the regulation of seed germination, and the transportation of defense-related molecules, were also altered. All these complex physiological and biochemical processes involve ABC transporters. In this study, comparing MT vs. CK, there was an enrichment of 10 differential metabolites in the ABC transporter pathway, of which 9 were upregulated. In ST vs. CK, three differential metabolites of the ABC transporter pathway were enriched, of which two were upregulated, suggesting that intercropping with other crops enhanced the ABC transporter pathway. In MT vs. ST, three different metabolites were enriched in the ABC transporter pathway, and their expression was upregulated. These results indicated that the ABC transporter pathway in the tobacco rhizosphere microenvironment would change when intercropped with other crops.

### 4.2 Differences in rhizosphere microbial species and functions between tobacco and different crops under intercropping

Rhizosphere microorganisms grow and colonize within the soil boundaries directly influenced by the plant root system. They mainly include bacteria, actinomycetes, fungi, algae, and protozoa, which are generally several to dozens of times more abundant than those outside the rhizosphere and actively interact with the plant root system (Mendes et al., 2013). The imbalance of rhizosphere microbiota has been shown to be an important reason for the occurrence of crop succession disorders, which affect the stability of the crop rhizosphere microbial community and negatively influence the crop root system, thus adversely affecting crop yield and quality (Ma et al., 2022; Hamid et al., 2017; Qin et al., 2022). This study showed that intercropping with other crops significantly increased the diversity and abundance of tobacco rhizosphere microorganisms: the number of microbial OTUs specific to the tobacco–maize and tobacco–soybean intercropping systems was significantly higher than that of the tobacco monoculture. At the phylum level, the three most dominant species were the same in all treatments: *Proteobacteria, Actinobacteria*, and *Acidobacteria*. Reganold and Wachter (2016) showed that different land use and cropping practices significantly affected the soil microbial community structure. In this study, while the three most dominant bacterial phyla were the same, the fourth-ranked dominant phylum was different in each treatment. The fourth-ranked phylum in abundance in tobacco monoculture and tobacco–soybean was *Gemmatimonadetes*, while in tobacco–maize, it was *Chloroflexi*. *Chloroflexi* is a widely distributed bacterial taxon comprising numerous classes found in various biosphere habitats. *Chloroflexi* comprises nine phyla but contains only 56 species (Hugenholtz and Stackebrandt, 2004). Chloroflexi are widely distributed in various biosphere environments and have been found in habitats such as soils, oceans, submarine hydrothermal zones, terrestrial hot springs, groundwater, activated sludge, and compost piles. They are involved in the biogeochemical cycling of elements such as C, N, and S, and they are important participants in the ecological processes in these environments (Liu et al., 2022; Spieck et al., 2020; Narsing Rao et al., 2022). In this study, through the metagenomic analysis of rhizosphere microorganisms under different intercropping systems, KEGG functional pathways enriched in the tobacco rhizosphere were found to be different. The number of functional pathways specific to tobacco–maize intercropping and tobacco monoculture was 5, while the number of functional pathways specific to tobacco soybean was only 1. When comparing the mean relative abundance of the same function across different treatments, it was discovered that the function K01992 was significantly more abundant in the tobacco–maize treatment compared to the other treatments. K01992 is related to the ABC-2 type transport system permease protein. The ABC transporter permease is the transmembrane subunit (TM) found in periplasmic binding protein (PBP)-dependent ABC transporters. These transporters consist of one PBP, two TMs, and two cytoplasmic ABC ATPase subunits and are mainly involved in importing solutes from the environment (Wang et al., 2023). The results of this study showed that tobacco and maize intercropping enhanced the ABC transporter pathway and transmembrane transport of plant secondary metabolites in tobacco roots. Plants produce many secondary metabolites such as alkaloids, terpenoids, and phenols, and ABC transporter proteins highly regulate the accumulation and excretion of these compounds. Tobacco and maize intercropping enhanced the transmembrane translocation of plant secondary metabolites between tobacco roots.

### 4.3 Different crops have varying effects on the ABC transporter pathway in the tobacco rhizosphere, influencing the regulation of related metabolites and genes

ABC transporters are a class of transmembrane transporter proteins commonly found in prokaryotes and eukaryotes and forming a large and functionally diverse protein family. The Arabidopsis genome encodes approximately 130 ABC transporter proteins, which are widely located in the plasma membrane, plastids, mitochondria, vesicles, endoplasmic reticulum, peroxisomes, and other locations of the cell and play key roles in phytohormone transport, lipid metabolism, detoxification of exogenous toxins, and disease resistance of plants (Liu, 2019; Ingle, 2011). To date, 49 ABC transporter proteins have been identified in the *Homo sapiens* genome, approximately 30 ABC transporter proteins have been identified in *Saccharomyces cerevisiae*, and 79 members of the ABC transporter protein family have been identified in *Escherichia coli* (Hwang et al., 2016). Compared with other organisms, the number of plant ABC transporter proteins is relatively high, with more than 130 members identified in *Oryza sativa* and *Arabidopsis thaliana*, which may be the result of plants’ adaptation to complex environments over a long period of time in the evolutionary process (Lefèvre et al., 2015). In this study, the introduction of intercropping increased complexity in the tobacco rhizosphere, which, in turn, influenced the functions of the ABC transporter pathway. This study showed that intercropping maize and soybeans could upregulate the overall expression of ABC transporters in the tobacco rhizosphere. The levels of L-histidine and L-valine were downregulated in the tobacco rhizosphere under both maize and soybean intercropping. L-histidine is involved in several metabolic pathways, such as the biosynthesis of plant secondary metabolites, biosynthesis of alkaloids derived from histidine and purine, metabolic pathways, and biosynthesis of amino acids. L-valine is involved in pantothenate and CoA biosynthesis, biosynthesis of secondary metabolites, biosynthesis of cofactors, protein digestion and absorption, and mineral absorption. Petersen et al. (2010) showed that histidine (His) plays a critical role in plant growth and development, both as one of the standard amino acids in proteins and as a metal-binding ligand. In the histidine metabolic pathway, intercropping with other crops upregulated the expression of *HisJ, HisM, HisQ*, and *HisP* genes. *HisP* is required for ATP hydrolysis, *HisQM* does not hydrolyze ATP, *HisP* is dependent on *HisQM* to deliver the induction signals from the soluble receptor *HisJ*, and *HisQM* regulates the ATPase activity of *HisP* (Liu and Ames, 1998). In another metabolic pathway, the abundance of genes in the branched-chain amino acid pathway, *LivK, LivH, Livg, LivM*, and *LivF*, was significantly higher than that of other genes. Leucine is transported into *E. coli* by two osmotic shock-sensitive, high-affinity systems (LIV-I and leucine-specific systems) and one membrane-bound, low-affinity system (LIV-II) (Landick et al., 1980). In *E. coli*, *LivJ* is part of the LIV system that is responsible for the transport of branched-chain amino acids, such as leucine, isoleucine, and valine (LIV), into the bacterial cell (Ribardo and Hendrixson, 2011). *LivK* could play a possible role in the signal sequence in determining the conformation of the binding protein precursor recognized by the membrane (Oxender et al., 1980). This study showed that different intercropped crops regulate metabolites in the tobacco rhizosphere microenvironment by modulating the upregulation of the expression of key genes in the phosphate and amino acid transporters pathway, thereby accelerating transmembrane transport between the soil and the tobacco rhizosphere. Analyzing the mineral and organic ion transporters pathway in the ABC transporter pathway, it was found that different intercropping combinations had different abilities to regulate the ABC transporter pathway. Moreover, in the Glycine betaine/Proline pathway, the relative abundance of *ProX*, *Prow*, and *Prov* was increased. Still, the degree of upregulation differed significantly among different intercrops. The three genes were found in the following order of abundance in the different treatments: MT > ST > CK. This indicated that maize intercropping has the capacity to more significantly activate the Glycine betaine/Proline pathway. Khalid et al. (2022) showed that the exogenous application of proline enhances growth and other physiological characteristics, upregulates osmoprotection functions, protects the integrity of the plasma lemma, reduces lipid peroxidation, and increases photosynthetic pigments, phenolic acids, flavonoids, and amino acids. Similarly, the foliar application of glycine betaine improves growth; upregulates osmoprotection functions; protects the integrity of the plasma lemma; reduces lipid peroxidation; increases photosynthetic pigments, phenolic acids, flavonoids, and amino acids; and enhances stress tolerance, carbon fixation, and leaf nitrogen content. Moreover, the foliar application of glycine betaine increased the relative water content, net photosynthetic rate, and catalase activity; decreased photorespiration ion leakage and lipid peroxidation; protected the oxygen-evolving complex; and prevented chlorosis. The results of this study showed that, among the two crops, maize and soybean, tobacco planted with maize could better promote the production and transportation of phenolic acids, flavonoids, and other bioactive compounds in the root system of tobacco, which could aid tobacco in combating abiotic stresses.

## 5 Conclusion

In this study, we analyzed the rhizosphere of tobacco in intercropping with different crops using metagenomics and metabolomics analysis. The results of this study showed that the contents of huperzine b, chlorobenzene, and P-chlorophenylalanine in tobacco rhizosphere soils differed significantly between the soybean–tobacco and maize–tobacco intercropping systems. Chlorobenzene and P-chlorophenylalanine had the highest relative abundance in the soybean–tobacco intercropping system and huperzine b in the maize–tobacco cropping system. The dominant microbial species in the tobacco rhizosphere did not differ significantly under different intercropping systems, except for minor differences in the proportions of individual dominant species. After intercropping with other crops, more metabolites associated with the ABC transporters pathway were enriched in the tobacco rhizosphere, which improved the transmembrane transport in the tobacco rhizosphere and improved tobacco resistance to abiotic stresses. This may be one of the mechanisms by which intercropping breaks the continuous cropping obstacle in tobacco.

## Data Availability

The datasets presented in this study can be found in online repositories. The names of the repository/repositories and accession number(s) can be found in the article/supplementary material.
